# Exceptional properties of hyper-resistant armor of a hydrothermal vent crab

**DOI:** 10.1038/s41598-022-15982-1

**Published:** 2022-07-12

**Authors:** Boongho Cho, Dongsung Kim, Taewon Kim

**Affiliations:** 1 Program in Biomedical Science and Engineering, Inha-ro, Michuhol-gu, Incheon, 22212 Republic of Korea; 2 Department of Ocean Sciences, Inha University, 100 Inha-ro, Michuhol-gu, Incheon, 22212 Republic of Korea; 3 Marine Ecosystem Research Center, Korea Institute of Ocean Science and Technology, 385, Haeyang-ro, Yeongdo-gu, Busan Metropolitan City, 49111 Republic of Korea

**Keywords:** Biomaterials, Biomimetics, Marine biology, Biomedical materials

## Abstract

Animals living in extreme environments, such as hydrothermal vents, would be expected to have evolved protective shells or exoskeletons to maintain homeostasis. The outer part of the exoskeleton of vent crabs (*Austinograea* sp.) in the Indian Ocean hydrothermal vent was one of the hardest (approximately 7 GPa) biological materials ever reported. To explore the exoskeletal characteristics of vent crabs which enable them to adapt to severe environments, a comparative analysis was conducted with the Asian paddle crab (*Charybdis japonica*) living in coastal areas. Nanoindentation, thermogravimetric analysis, scanning electron microscopy, energy dispersive x-ray analysis, and Raman spectroscopy were used to analyze the mechanical properties, thermal stability, structure, surface components, and the composition of compounds, respectively. Though both species have four-layered exoskeletons, the outermost layer of the vent crab, a nano-granular structure, was much thicker than that of the coastal crab. The proportions of aluminum and sulfur that constitute the epicuticle of the exoskeleton were higher in the vent crab than in the coastal crab. There was a lack of water or volatile substances, lots of CaCO_3_, and no carotenoid-based compounds in the exoskeleton of the vent crab. These might have improved the mechanical properties and thermal stability of the hydrothermal species.

## Introduction

Characteristics of deep-sea organisms have been receiving increasing attention for bioinspired materials science, biomineralization, and biomimetrics^1–4^. The hard outer layer of animals such as exoskeletons, shells, teeth, and scales is a topic of high interest^5–12^. In an extreme environment such as a hydrothermal vent^13^, it could have the function of protecting the organisms from both fluctuating heat from 0 to over 400 °C and pressure. Yao et al.^4^ investigated the protection mechanisms of the iron-plated armor in a gastropod living in the hydrothermal vent in the Indian Ocean. Cho et al.^14^ found unique exoskeletal characteristics of the bythograeid crab *Austinograea rodriguezensis* living in a hydrothermal vent, which might have evolved to survive in extreme environments. Although several studies have been conducted on the evolutionary (due to pressure and thermal) aspects of exoskeletons using comparative analysis of different habitats to determine the survival mechanisms^15–18^, there has been no comparative research on the aspect of both pressure and temperature.

**Figure 1:**
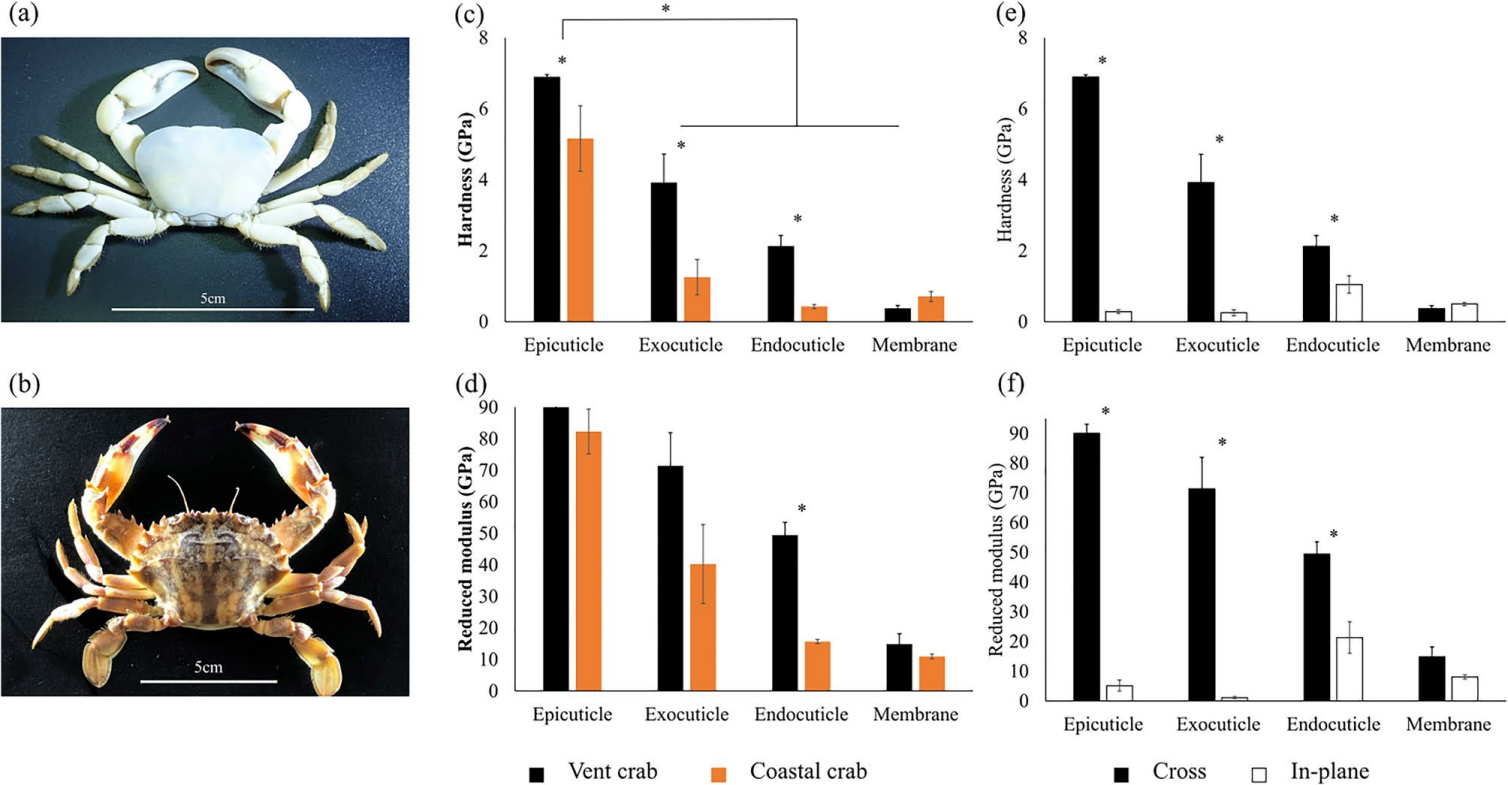
Crab samples and mechanical properties. The samples of the two species [(**a**) *Austinograea* sp., (**b**) *C. japonica*]. (**c**,**d**) The hardness and reduced modulus of the exoskeletons of the two crabs in cross-sections (mean ± SE). (**e**,**f**) The hardness and reduced modulus of the vent crab in cross-sections and in-plane sections^14^ (mean ± SE) [significant difference is indicated by an asterisk (*)].

To explore the exoskeletal characteristics of vent crabs (*Austinograea* sp.) (Fig. 1a) that enable them to adapt to severe environments in the hydrothermal vent, we conducted the first-time comparative analysis focusing on components with the Asian paddle crab (*Charybdis japonica*) (Fig. 1b) living in coastal areas. In addition, we intended to confirm the difference in the evolutionary characteristics of the exoskeleton according to the differences in the environmental factors between the two different habitats. We hypothesized that vent crabs might have evolved special components in their exoskeletons to adapt to deep-sea hydrothermal vents. Accordingly, we predicted that the exoskeleton properties (mechanical properties and thermal stability) of the vent crab would be better than those of the coastal crabs. Then, we investigated the exoskeletal structure and components of the vent crab to reveal what made the exoskeleton of vent crabs superior to that of the shore crabs.

## Results

### Mechanical properties

**Figure 2:**
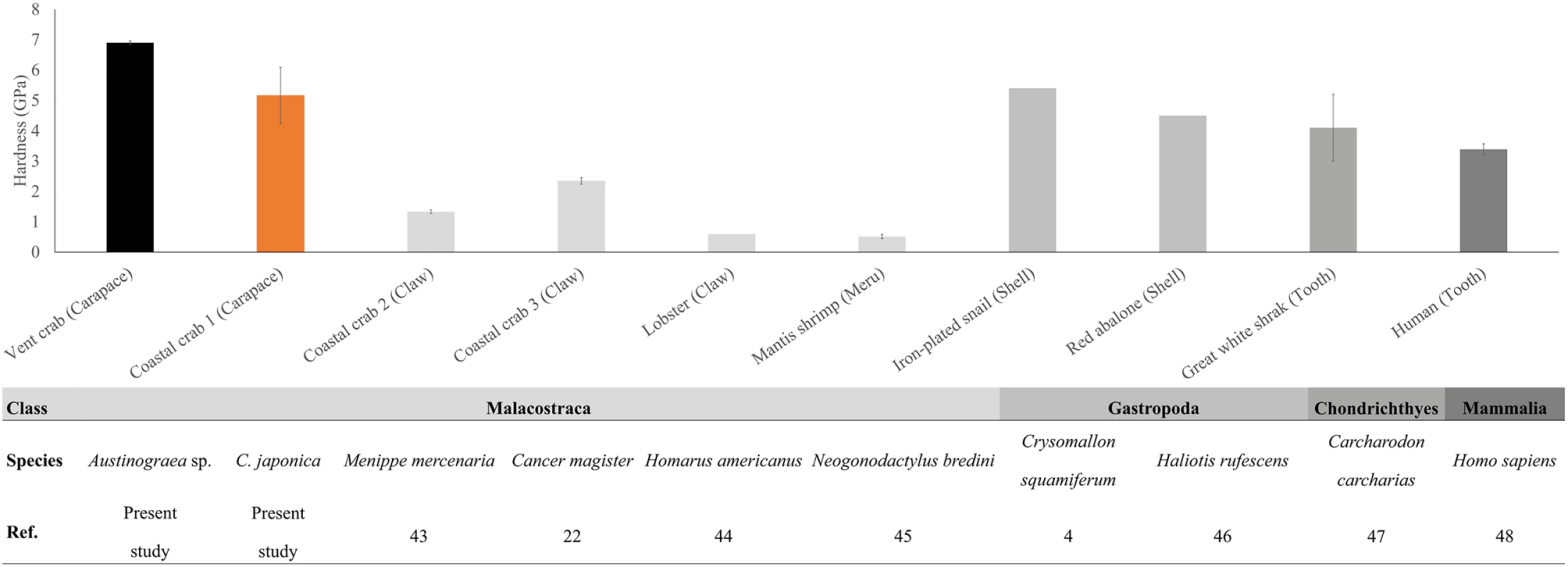
Comparison of the hardness for various species (mean ± SE)^43–48^.

The hardness was significantly different between the four layers in both species (One-way ANOVA; vent crab: *F*_3,11_ = 41.547, *p* < 0.0001, coastal crab: *F*_3,11_ = 14.685, *p* < 0.001) and that of the epicuticle was higher than that of other layers (Tukey post hoc test, *p* < 0.05) (Fig. 1c). Among the mechanical properties of the exoskeleton, the hardness of all layers was significantly higher in the vent species than in the coastal species except for the membrane layer (Mann–Whitney *U*-test; epicuticle: *W* = 6.000, *p* = 0.05, *t* test; exocuticle: df = 4, *p* = 0.024 and endocuticle: df = 2.152, *p* = 0.013), and the reduced modulus was significantly higher in the endocuticle layer in the vent species than in the coastal species (df = 0.012, *p* = 0.006) (Fig. 1c, d). The mechanical properties of the cross-section of the vent crab exoskeleton were significantly higher than the in-plane section except for the membrane layer (hardness of epicuticle: df = 4, *p* < 0.0001, hardness of exocuticle: df = 2.027, *p* = 0.023, hardness of endocuticle: df = 4, *p* = 0.025, reduced modulus of exocuticle: df = 2.004, *p* = 0.011, reduced modulus of endocuticle: df = 4, *p* = 0.007, Mann–Whitney U-test; reduced modulus of epicuticle: W = 6.000, *p* = 0.05) (Fig. 1e, f). Such hardness of the vent crab was the strongest among those of hardest parts of various species from previous studies (Fig. 2).

### Thermal stability

**Figure 3:**
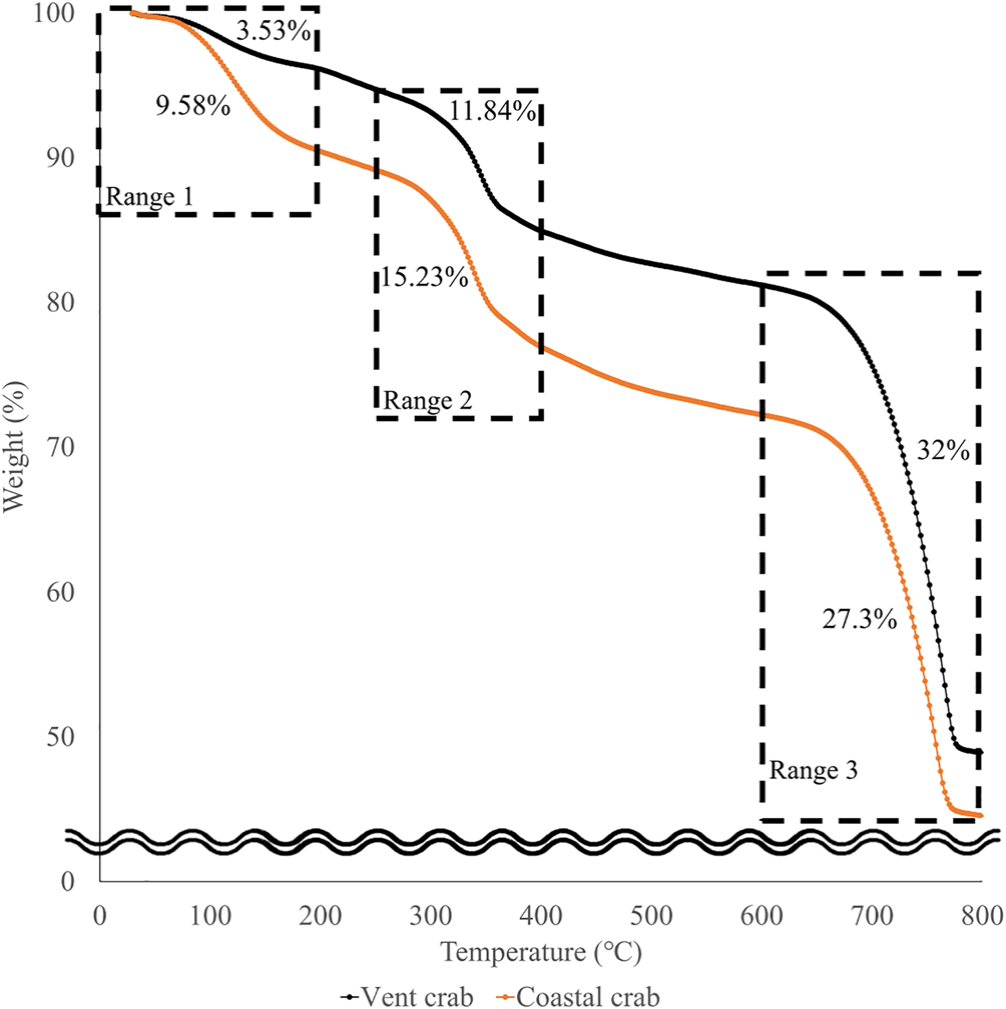
Thermogravimetric analysis (TGA) curve. The first stage of weight loss was visible from room temperature to 200 °C, which indicates the removal of water or volatile components. The second weight loss (250–500 °C) indicates that chitin or organic matter has been removed. The third weight loss (600–800 °C) indicates that calcium carbonate, an inorganic component that forms the exoskeleton, has been removed.

Thermogravimetric analysis (TGA) results showed that weight loss occurred in three stages (Fig. 3 and Supplementary Table S1). There was a significant difference between two species with respect to weight loss in all three stages (*t* test; first stage: df = 4, *p* = 0.005, and second stage: df = 4, *p* = 0.046, Mann–Whitney *U*-test; third stage: *W* = 6.000, *p* = 0.05) (Fig. 3 and Supplementary Table S1). The weight loss (%) was significantly lower in the vent species than in the coastal species (vent crab: 50.9 ± 1.32 and coastal crab: 55.19 ± 0.57, df = 4, *p* = 0.011).

### Characteristics of structure

The exoskeletons of both species were composed of four layers: epicuticle, exocuticle, endocuticle, and membrane layer. Each layer had a specific structure (Supplementary Fig. S1a, f). The outermost layer, the epicuticle had a nano-granule structure (size of the granule diameter (nm): vent: 14.95 ± 1.34, coastal: 16.91 ± 0.63; Supplementary Fig. S1b, g). The second and third layers were made with a Bouligand structure (Supplementary Fig. S1c, d, h, i). The innermost layer had a multilayer structure (Supplementary Fig. S1e, j). The characteristics of each structure were similar between the two species. However, the ratio of a thickness (%) of the epicuticle was much higher in the vent crab than in the coastal crab by about 248.39% (vent crab: 0.77 ± 0.11 and coastal crab: 0.31 ± 0.06, df = 4, *p* = 0.022). The ratio of the thickness (%) of the exocuticle, endocuticle, and membrane was 13.4 ± 0.84, 83.92 ± 0.85, and 1.91 ± 0.48 respectively in vent crabs, and 15.44 ± 2.6, 82.17 ± 2.93, and 2.09 ± 0.39 respectively in coastal crabs.

### Component characteristics

**Figure 4:**
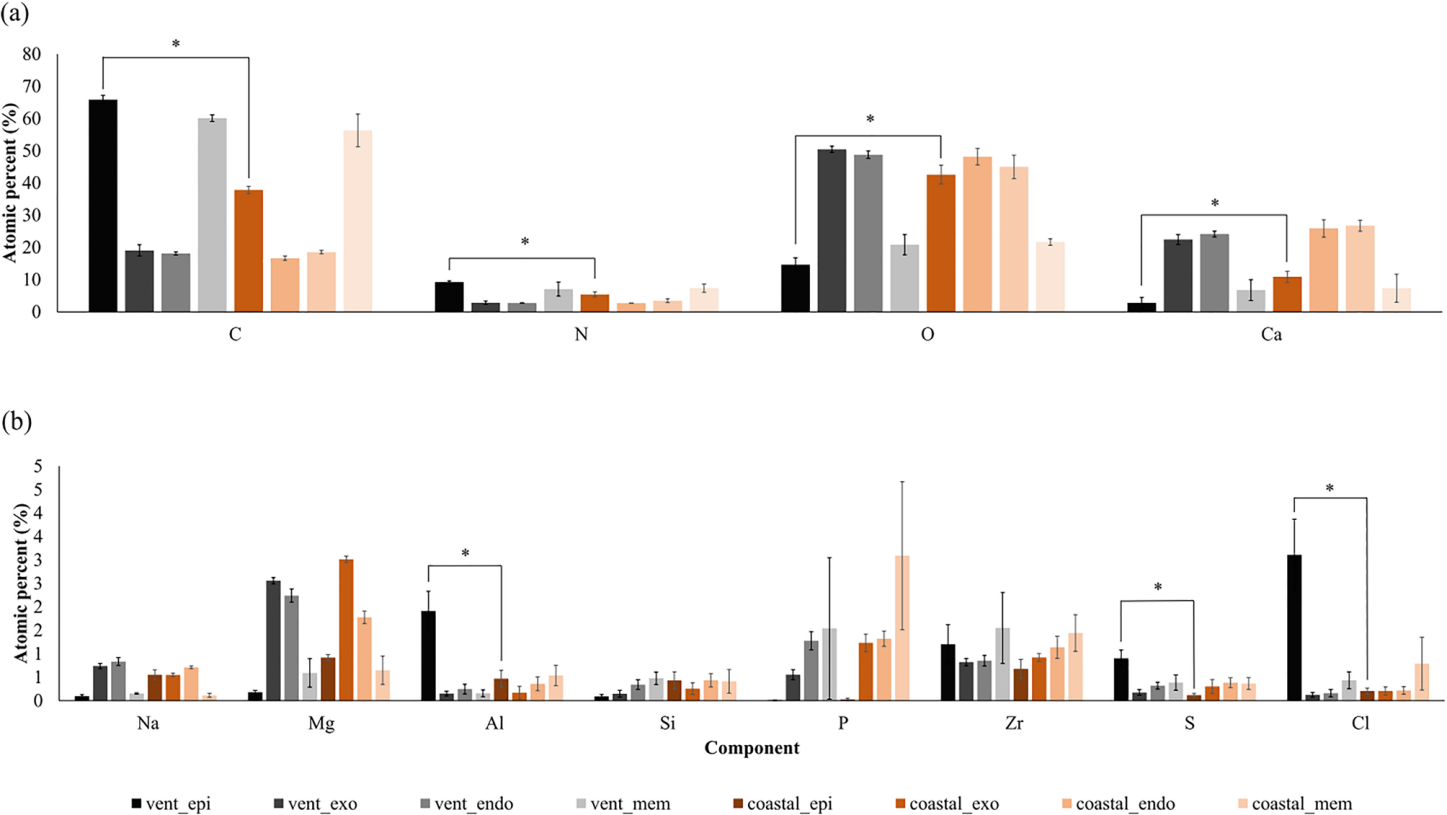
Component analysis results using EDX. Comparison of (**a**) main and (**b**) minor elements of the exoskeleton between the two crabs [significant difference is indicated by an asterisk (*)] (mean ± SE).

Energy dispersive x-ray analysis (EDX) showed that exoskeletons of both crab species consisted of 12 elements, but the distribution of these components was different between the two species (Fig. 4). The main elements were carbon, nitrogen, oxygen, and calcium. When the main elements between the two species, in the first layer, the epicuticle layer, were compared, all four elements showed significant differences (Fig. 4a). In the epicuticle layer, the vent crab had more carbon and nitrogen, while the coastal crab had more oxygen and calcium (Mann–Whitney *U*-test; carbon: *W* = 6.000, *p* = 0.05, *t* test; nitrogen: df = 4, *p* = 0.012, oxygen: df = 4, *p* = 0.002, and calcium: df = 4, *p* = 0.03), and the rest of the 8 minor elements, including aluminum, sulfur, and chlorine were higher in vent crabs than in coastal crabs (aluminum: df = 4, *p* = 0.034, sulfur: df = 4, *p* = 0.012, and chlorine: df = 4, *p* = 0.019) (Fig. 4b). Raman spectroscopy revealed that the vent crab comprised calcite, calcium phosphate, ring breathing (protein), and organic material (α-chitin). Meanwhile, the coastal crab had a variety of carotenoid-based compounds [β-carotene and proteins, unsaturated fatty acids, and astaxanthin (ATX)] (Supplementary Fig. S2, and Supplementary Table S2).

## Discussion

Comparison between the two species revealed that the hardness of the exoskeleton in the vent crab (*Austinograea* sp.) was much higher than that in the coastal crab (*C. japonica*)**.** In particular, the epicuticle was the hardest part of the vent crab’s exoskeleton. Indeed, it is one of the hardest biological materials ever reported compared with the hard parts (e.g., exoskeletons, shells, and teeth) of other species such as crustaceans, mollusks, and fish. Furthermore, the thermal stability of the exoskeleton in the vent crab was much higher than that in the coastal crab as expected.

There could be two main reasons how to vent crab has acquired superior hardness. First, the epicuticle layer, which has high fracture resistance and excellent energy dissipation from external threats^4^, is thicker than that of the coastal crab. In addition, the contents of aluminum and sulfur elements constituting the epicuticle are significantly higher than those of coastal species. Aluminum existed in the form of a gel in the exoskeleton of amphipods living in the Mariana Trench, which is beneficial to withstand high pressure^15^. Sulfur could also play a role in improving the mechanical properties as shown in gastropod shells living in deep-sea hydrothermal vents^4^ and calcium sulfate bonded with fluorapatite^19^. The reason for the high content of aluminum in the exoskeleton may be a characteristic of the seawater around the hydrothermal vents^20^. Also, it is possible that the aluminum and sulfur contained in the exoskeleton of the vent crab could be forced biomineralization^1^ and the mechanical performance was improved due to the crystallinity of the exoskeleton affected by the hydrothermal environment^21^.

In a previous study on the exoskeleton of *A. rodriguezensis*, the estimated values of the mechanical properties of the endocuticle layer were superior to those of the epicuticle layer^14^. The major difference between our present study and the previous study is probably due to the mechanical anisotropy of the exoskeleton. Lian and Wang^22^ studied the differences in the mechanical properties of the cross-section and in-plane section, and they revealed that the mechanical properties of the in-plane section could play a role as an indicator of resistance to external threats. The results of this study suggest that the reason in the mechanical properties of the cross-section is higher than those of the in-plane section may be due to resistance to other factors (temperature or pressure) rather than resistance to external intrusion, a topic that requires further study.

The exocuticle and endocuticle layers are made with a Bouligand structure, which is known to prevent fractures^23^. The maximum thickness of one layer of the Bouligand structure (MTOB), known to have a negative correlation with hardness^22^, was significantly higher in the vent crab than in the coastal crab (27.91 ± 1.12 μm for vent species and 20.62 ± 1.7 μm for coastal species). Even though the MTOB in the endocuticle of the vent crab was thicker than that of the coastal crab, however, the endocuticle of the vent crab had significantly higher hardness than that of the coastal species. Such an MTOB may not greatly affect the mechanical properties.

In the first stage of weight loss after TGA, where the difference in weight loss (%) was the greatest between the two species, the vent crabs showed a reduction in weight by approximately 40.61% of the coastal species. This effect was due to the degradation of water from hydrophilic groups in chitin chains constituting the exoskeleton^24–27^. The second stage corresponds to the decomposition of chitin a representative organic material in the exoskeleton. In this stage, only chitin was decomposed in the vent crab, but unsaturated fatty acids (220–365 °C) constituting carotenoid-based compounds were also decomposed along with chitin in the coastal species. ATX is a yellow to red color pigment^28^, commonly distributed in the exoskeleton of coastal crustaceans, and its main roles include camouflage^29^ and reproduction^30^. The reason of absence carotenoid-based compounds in the exoskeleton of the vent crab might be that they do not need the ATX in the absence of light and predators^31^. The fact that they lost their eyes also suggests that they do not use visual communications. Carotenoid-based compounds are found in the organs and tissues of some hydrothermal crustaceans^32^, but not in the exoskeleton of them. These are not synthesized in most animals^32,33^, but provided as a dietary supplement^34,35^. Changes in the ecological position and diet of the crabs with habitat have resulted in differences in the organic compounds that make up the crab exoskeleton, which may affect the thermal stability of the exoskeleton. The third stage represents the calcium carbonate constituting the exoskeleton, which is decomposed from CaCO_3_ to CaO and CO_2_^26^. Exoskeletal calcium carbonate interferes with the pronouncement of chitin amides I and II, thus inhibiting the thermal stability of chitin^26^. Although the hydrothermal species contains a high proportion of calcium carbonates, the thermal stability of the vent crab exoskeleton is greater than that of the coastal species. This result may be due to the difference in the ratio of chitin and calcium carbonate constituting the crustacean exoskeleton.

## Conclusion

By comparing the mechanical and thermal characteristics with the exoskeletons of coastal crabs, we found extraordinary structural and component features in the armor of a hydrothermal vent crab. The exceptional hardness of the exoskeleton is probably due to the epicuticle’s thick and nano-granular structure, containing higher amounts of aluminum and sulfur components. Higher thermal stability in the vent crab exoskeleton can be attained by a high proportion of CaCO_3_ and losing volatile substances and carotenoid-based pigment compounds. These armor characteristics might have assisted the crabs to survive in extreme environments with high pressures and temperatures. The exceptional properties of the crab exoskeleton can be applied to novel technologies to make artificial skeletons for medical applications and protective gears such as structural firefighting clothes. It is expected to be take a center stage as a compelling biological material for biological engineering and extreme biomimetics^36^.

## Materials and methods

### Sample preparation

The sampling of vent crabs (*Austinograea* sp.)^37^ was conducted in the Onnuri vent field (depth: 2014.5 m) in the Indian Ocean (latitude 11° 14′ 55.9″ S and longitude 66° 15′ 15.1″ E) using TeleVision-grab (TV-grab) on the Research Vessel (R/V) ISABU (Dive No. 4) on June 29, 2019. For comparative analysis, coastal crabs (*C. japonica*) were purchased on April 6, 2020, at Incheon Comprehensive Fish Market, Republic of Korea (latitude 37° 27′ 14.7″ N and longitude 126° 36′ 23.3″ E). Coastal species have a close phylogenetic relationship with the vent crabs. They are in the same subsection and are branched from superfamily. The sizes of the vent and coastal crab specimens (*N* = 3) were 3.42 cm ± 0.02 cm and 5.74 cm ± 0.03 cm [mean ± standard error (SE)], respectively. The samples were stored in 75% ethanol^38^.

The flattest part of the carapace was divided into five pieces (length and width: 0.25 cm) using digital calipers (CD-15PSX, Mitutoyo, Kanagawa, Japan). Each sample was separately used for five analyses [nanoindentation, thermogravimetric analysis (TGA), scanning electron microscopy (SEM), energy dispersive x-ray analysis (EDX), and Raman spectroscopy]. The samples were washed with distilled water and the tissue or muscle was removed using a soft brush^39,40^. The samples for nanoindentation, EDX, and Raman spectroscopy were fixed in cold resin to avoid heating during the hardening procedure. All the surfaces of the resin samples were polished using a 5 μm particle sized sandpaper. The sample for TGA was filtered through a 500 μm mesh sieve to remove small contaminants^41^. The sample for the SEM analysis was prepared using the fracturing method^40^. It could protect the microstructure of the exoskeleton from abrasion by knife or polishing.

### Properties analysis

The analysis consisted of four main stages: (1) analysis of the mechanical properties, (2) analysis of the thermal stability, (3) structural analysis, and (4) component analysis. The mechanical properties were measured using a nanoindenter (G200, KLA, California, United States of America) to determine the hardness and reduced modulus of each layer of the exoskeleton (force: 0.051 gf, peak hold time: 10 s, time to load: 10 s). TGA (STA 409 PC, NETZSCH, Selb, Deutschland) was used to quantitatively determine the thermal stability of the exoskeleton (heating rate: 10 °C/min, atmosphere: nitrogen gas)^24,25,42^. The weight loss (%) after burning up to 800 °C is the thermal stability evaluation index^26^. For structural analysis, SEM (S-4300SE, Hitachi, Ltd, Tokyo, Japan) was used to identify the structure of the entire cross-section of the exoskeleton and the microstructure of each layer. The samples were coated with platinum for 120 s. Two approaches were used for the component analysis. EDX (S-4300SE, Hitachi, LTD, Tokyo, Japan) was used for elemental component analysis [coating material: platinum (Pt), coating duration: 20 s] and Raman spectroscopy (LabRAm HR Evolution, HORIBA, Ltd, Kyoto, Japan) was used to identify compounds at the cross-section surface of the exoskeleton.

### Statistical analysis

A one-way ANOVA was used to compare the hardness of each layer of the exoskeleton for each species. Tukey’s honest significant difference (HSD) test was used as a post hoc test to confirm that the epicuticle had a superior hardness than the other layers. We used a one-tailed independent *t* test for normally distributed data and the Mann–Whitney *U*-test for non-normally distributed data to test if there were significant differences in the mechanical properties (hardness and reduced modulus) and thermal stabilities (weight loss) of the exoskeleton between the species. The data used for the *t* test confirmed the equal variance by Levene's test. To compare the thickness and components of the exoskeleton by species, two-tailed independent *t* test and Mann–Whitney *U*-test were conducted. All statistical analyses were performed using SPSS software (version 19.0; SPSS, Inc., Chicago, USA). All datasets are presented as mean ± standard error (SE).

## Supplementary Information

Supplementary Information.

## Data Availability

The data that support the findings of this study are available from the corresponding author upon reasonable request.
