# Supplementary material for: Exceptional properties of hyper-resistant armor of a hydrothermal vent crab
Source: Sci Rep. 2022 Jul 12;12:11816. doi: 10.1038/s41598-022-15982-1 (PMC9276715; doi:10.1038/s41598-022-15982-1)
Supplement: Supplementary file 1 — Supplementary Information. [file 41598_2022_15982_MOESM1_ESM.docx]

**Supporting Information**

Title

Exceptional Properties of Hyper-resistant Armor of a Hydrothermal Vent Crab

**Author(s) information and affiliations**

Boongho Cho ^1,2^, Dongsung Kim ^3^, Taewon Kim ^1,2*^

- ^1^ Program in Biomedical Science and Engineering, Inha-ro, Michuhol-gu, Incheon 22212, Republic of Korea
- ^2^ Department of Ocean Sciences, Inha University, 100 Inha-ro, Michuhol-gu, Incheon 22212, Republic of Korea
- ^3^ Marine Ecosystem Research Center, Korea Institute of Ocean Science & Technology, 385, Haeyang-ro, Yeongdo-gu, Busan Metropolitan City 49111, Republic of Korea

**Corresponding Author^*^**

Taewon Kim, tel: 82-10-8726-3070, e-mail: ktwon@inha.ac.kr

- Supplementary Figures
-
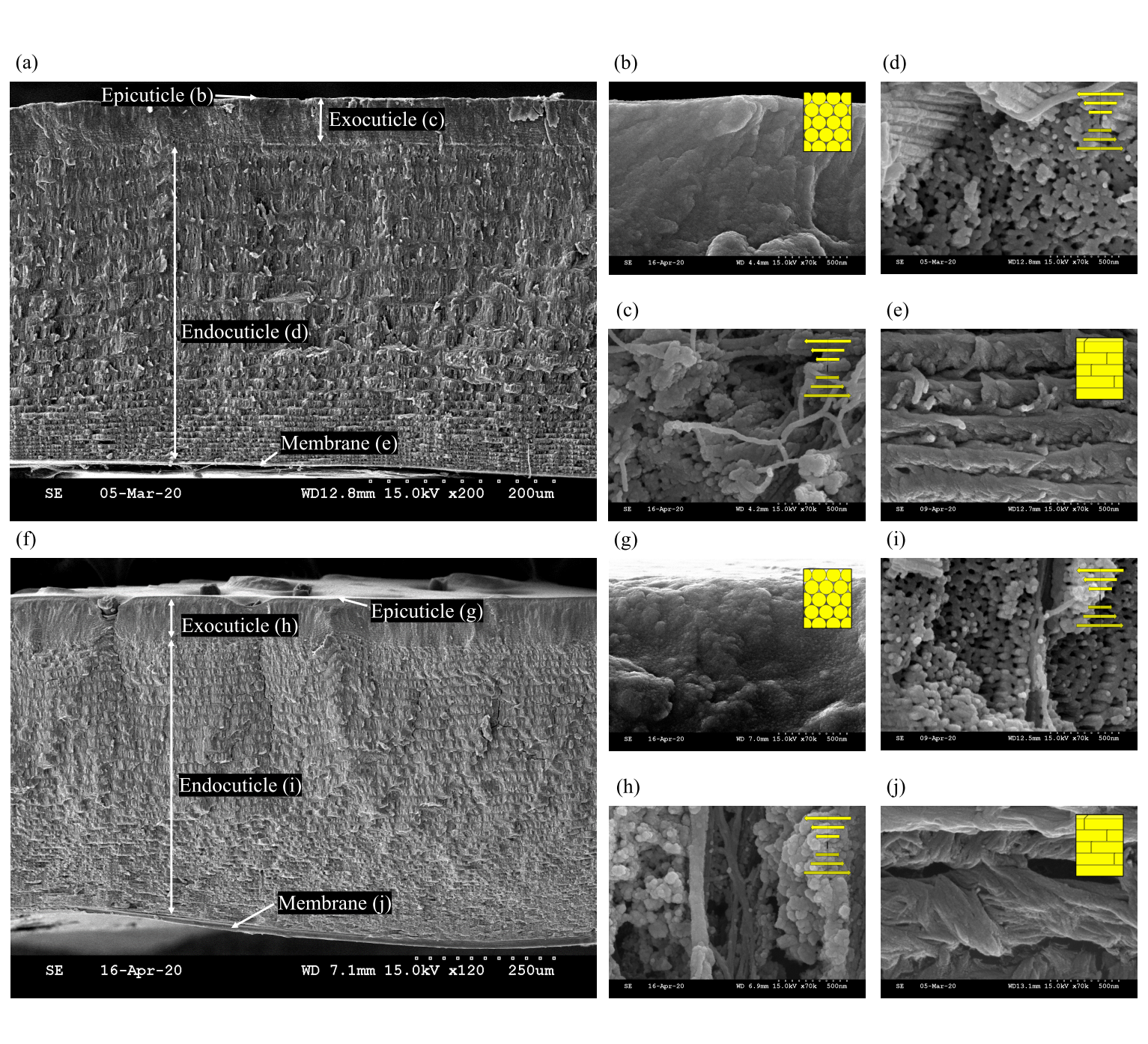


**Figure S1.** Cross-section of the exoskeleton of the (a) vent crab and (f) coastal crab. (b, g) The granular structure of the epicuticle. The Bouligand structure of the (c, h) exocuticle and the (d, i) endocuticle. (e, j) The multilayer structure of the membrane layer.

**
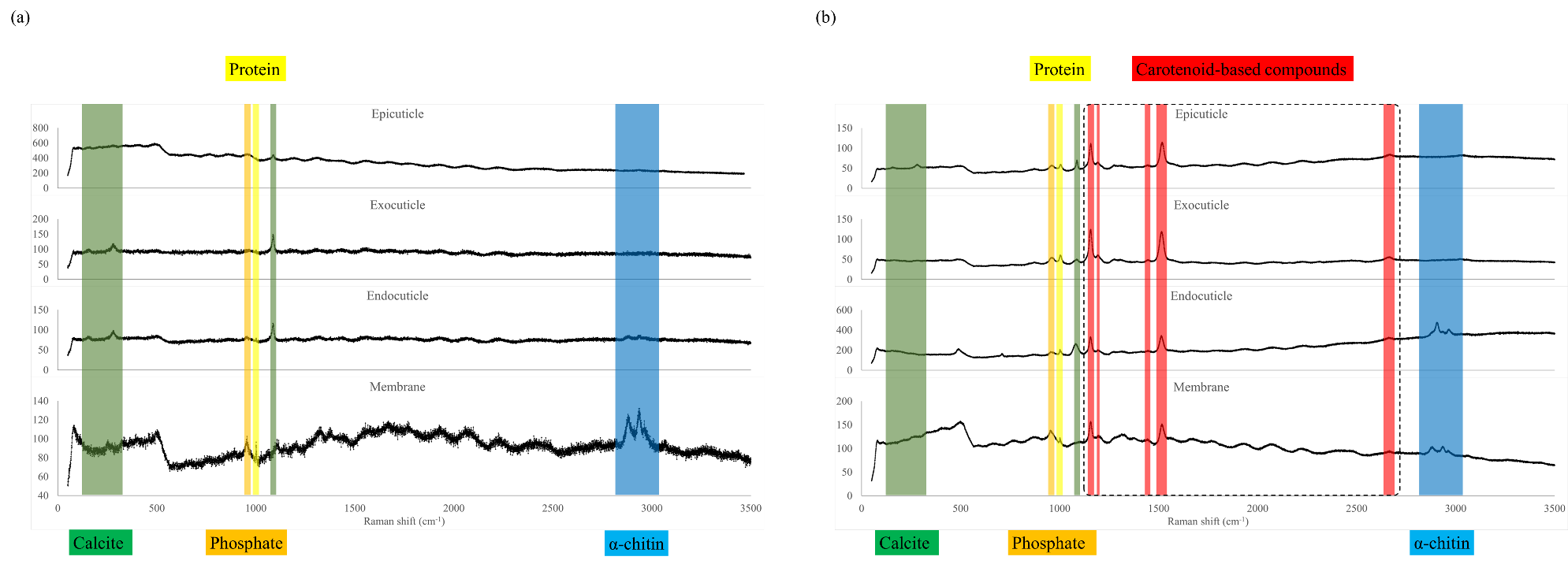
**

**Figure S2.** Raman spectra of exoskeleton [(a) vent crab and (b) coastal crab].

- Supplementary Tables

**Table S1.** Indicators related to the TGA results [significant difference is indicated by an asterisk (^*^)] (mean ± SE).

| **Stages** | **1** | | **2** | | | **3** | |
| --- | --- | --- | --- | --- | --- | --- | --- |
| **Species** | Vent crab | Coastal crab | Vent crab | | Coastal crab | Vent crab | Coastal crab |
| **Weight loss (%)** | *3.53 ± 0.28 | *9.58 ± 0.43 | *11.84 ± 0.45 | | *15.23 ± 1.8 | *32 ± 0.8 | *27.3 ± 0.57 |
| **Substances**  **and references** | Water^1–3^ ,  other volatile components^1,4^ | | Chitin^2,3,5^ | | | Calcium carbonate (CaCO_3_)^4–6^ | |
|  |  |  | - | ATX^7,8^  , Unsaturated fatty acid^9^ | |  |  |

**Table S2.** The peaks of the exoskeleton sample with the corresponding substances.

| Raman shift (cm^-1^) | Substances | References |
| --- | --- | --- |
| 151-154, 281-283, 710-713 | Calcite, amorphous calcium carbonate (ACC) | ^10–12^ |
| 954-956 | Phosphate | ^13^ |
| 1001-1002 | Ring breathing, protein | ^14–17^ |
| 1086-1090 | Calcite, ACC | ^10–13,18^ |
| 1154-1160 | ATX, α-chitin | ^13,19,20^ |
| 1192-1196 | ATX, β-carotene, amide III | ^19,20^ |
| 1274-1275 | Amide III | ^19^ |
| 1447-1449 | Unsaturated fatty acid | ^19^ |
| 1513-1517 | ATX, β-carotene, and related carotenoids | ^19,20^ |
| 2662-2671 | ATX | ^20^ |
| 2880-3020 | Organic material (α-chitin) | ^10^ |

**References**

1. Kaya, M. *et al.* On chemistry of γ-chitin. *Carbohydr. Polym.* **176**, 177–186 (2017).

2. Metin, C., Alparslan, Y., Baygar, T. & Baygar, T. Physicochemical, microstructural and thermal characterization of chitosan from blue crab shell waste and its bioactivity characteristics. *J. Polym. Environ.* **27**, 2552–2561 (2019).

3. Mendez‐Alpuche, A. A., Ríos‐Soberanis, C. R., Rodriguez‐Laviada, J., Perez‐Pacheco, E. & Zaldivar‐Rae, J. A. Physicochemical comparison of chitin extracted from horseshoe crab (*Limulus polyphemus*) exoskeleton and exuviae. *ChemistrySelect* **5**, 11745–11752 (2020).

4. Gbenebor, O. P., Adeosun, S. O., Lawal, G. I. & Jun, S. Role of CaCO3 in the physicochemical properties of crustacean-sourced structural polysaccharides. *Mater. Chem. Phys.* **184**, 203–209 (2016).

5. Romano, P., Fabritius, H. & Raabe, D. The exoskeleton of the lobster *Homarus americanus* as an example of a smart anisotropic biological material. *Acta Biomater.* **3**, 301–309 (2007).

6. Agrawal, S., Singh, B. & Sharma, Y. C. Exoskeleton of a mollusk (*Pila globosa*) as a heterogeneous catalyst for synthesis of biodiesel using used frying oil. *Ind. Eng. Chem. Res.* **51**, 11875–11880 (2012).

7. Yuan, C., Jin, Z., Xu, X., Zhuang, H. & Shen, W. Preparation and stability of the inclusion complex of astaxanthin with hydroxypropyl-β-cyclodextrin. *Food Chem.* **109**, 264–268 (2008).

8. Dong, S. *et al.* Inclusion complexes of astaxanthin with hydroxypropyl-β-cyclodextrin: Parameters optimization, spectroscopic profiles, and properties. *Eur. J. Lipid Sci. Technol.* **116**, 978–986 (2014).

9. Raba, D. N., Chambre, D. R., Copolovici, D.-M., Moldovan, C. & Copolovici, L. O. The influence of high-temperature heating on composition and thermo-oxidative stability of the oil extracted from Arabica coffee beans. *PLOS ONE* **13**, e0200314 (2018).

10. Hild, S., Marti, O. & Ziegler, A. Spatial distribution of calcite and amorphous calcium carbonate in the cuticle of the terrestrial crustaceans *Porcellio scaber* and *Armadillidium vulgare*. *J. Struct. Biol.* **163**, 100–108 (2008).

11. Borromeo, L. *et al.* Quick, easy, and economic mineralogical studies of flooded chalk for eor experiments using raman spectroscopy. *Minerals* **8**, 221 (2018).

12. Tao, J., Zhou, D., Zhang, Z., Xu, X. & Tang, R. Magnesium-aspartate-based crystallization switch inspired from shell molt of crustacean. *Proc. Natl. Acad. Sci.* **106**, 22096–22101 (2009).

13. Bentov, S., Aflalo, E. D., Tynyakov, J., Glazer, L. & Sagi, A. Calcium phosphate mineralization is widely applied in crustacean mandibles. *Sci. Rep.* **6**, 22118 (2016).

14. Esmonde-White, K. A. *et al.* Raman spectroscopy of synovial fluid as a tool for diagnosing osteoarthritis. *J. Biomed. Opt.* **14**, 034013 (2009).

15. Li, Y. *et al.* Rapid detection of nasopharyngeal cancer using Raman spectroscopy and multivariate statistical analysis. *Mol. Clin. Oncol.* **3**, 375–380 (2015).

16. Lasalvia, M., Perna, G. & Capozzi, V. Raman Spectroscopy of Human Neuronal and Epidermal Cells Exposed to an Insecticide Mixture of Chlorpyrifos and Deltamethrin. *Appl. Spectrosc.* **68**, 1123–1131 (2014).

17. Guimarães, A. E. *et al.* Near Infrared Raman Spectroscopy (NIRS): A technique for doping control. *Spectroscopy* **20**, 185–194 (2006).

18. Wang, D., Hamm, L. M., Bodnar, R. J. & Dove, P. M. Raman spectroscopic characterization of the magnesium content in amorphous calcium carbonates. *J. Raman Spectrosc.* **43**, 543–548 (2012).

19. Shao, Y., Gu, W., Jiang, L., Zhu, Y. & Gong, A. Study on the visualization of pigment in *Haematococcus pluvialis* by raman spectroscopy technique. *Sci. Rep.* **9**, 12097 (2019).

20. Nekvapil, F. *et al.* Color-specific porosity in double pigmented natural 3d-nanoarchitectures of blue crab shell. *Sci. Rep.* **10**, 3019 (2020).
